# Sequence Variation in the Bovine Lipin-1 Gene (*LPIN1*) and Its Association with Milk Fat and Protein Contents in New Zealand Holstein-Friesian × Jersey (HF × J)-cross Dairy Cows

**DOI:** 10.3390/ani11113223

**Published:** 2021-11-11

**Authors:** Xiaohua Du, Huitong Zhou, Xia Liu, Yunhai Li, Jonathan G. H. Hickford

**Affiliations:** 1 College of Veterinary Medicine, Gansu Agricultural University, Lanzhou 730070, China; duxh@gsau.edu.cn; 2 Gene-Marker Laboratory, Faculty of Agriculture and Life Sciences, Lincoln University, Lincoln 7647, New Zealand; Huitong.Zhou@lincoln.ac.nz (H.Z.); Yunhai.Li@Lincolnuni.ac.nz (Y.L.); 3 College of Life Science and Technology, Gansu Agricultural University, Lanzhou 730070, China

**Keywords:** *LPIN1*, bovine, milk fat percentage, milk protein percentage, PCR-SSCP

## Abstract

**Simple Summary:**

Lipins are a family of proteins involved in lipid metabolism through their phosphatidate phosphatase and transcriptional co-activator activities. Of the three family members identified, lipin-1 is highly upregulated in the bovine mammary gland during lactation, and has been implicated in regulating milk production, albeit with inconsistent results in different breeds of cattle in studies. Accordingly, its role in regulating milk synthesis is not well understood. In this study, we used polymerase chain reaction-single strand conformation polymorphism (PCR-SSCP) analyses to investigate variation in the bovine lipin-1 gene in New Zealand Holstein-Friesian × Jersey-cross dairy cows and found that variation in the sixth coding exon was associated with milk fat percentage and protein percentage. This suggests that lipin-1 regulates the synthesis of milk fat and milk protein.

**Abstract:**

Lipin-1 is known to play a regulatory role in tissues that function in lipid metabolism. In dairy cows, the lipin-1 gene (*LPIN1*) is highly expressed in the mammary gland, but its function in milk production is less understood. In this study, we used PCR-single strand conformation polymorphism analysis to investigate sequence variation in three regions of bovine *LPIN1* in New Zealand Holstein-Friesian × Jersey (HF × J)-cross dairy cows, including part of the 5′ non-coding region, the region containing the *LPIN1β*-spliced exon, and the sixth coding exon that encodes the putative transcriptional activating domain of the protein. No variation was found in the *LPIN1β*-spliced exon, but two sequence variants containing one single nucleotide polymorphism (SNP) were identified in the 5′ non-coding region and four sequence variants containing four non-synonymous SNPs were identified in the sixth coding exon. Among the three common variants of the sixth coding exon, variant *C* was found to be associated with an increase in milk fat percentage (presence 4.96 ± 0.034% vs. absence 4.81 ± 0.050%; *p* = 0.006) and milk protein percentage (presence 4.09 ± 0.017% vs. absence 3.99 ± 0.025%; *p* = 0.001), but no associations (*p* > 0.01) were detected for milk yield. These results suggest that variation in *LPIN1* affect the synthesis of fat and proteins in milk and has potential as a gene-marker to improve milk production traits.

## 1. Introduction

Lipins are phosphatidate phosphatase (PAP) enzymes and catalyse the dephosphorylation of phosphatidic acid to form diacylglycerol (DAG), the substrate for triglyceride (TG) [1]. Three members of the lipin family have been identified in mammals, lipin-1, lipin-2, and lipin-3, and each exhibit distinct tissue expression patterns [2,3]. Using two different transgenic mouse strains, Phan and Reue [4] demonstrated that enhanced lipin expression in either adipose tissue, or skeletal muscle, promotes obesity. Lipin levels in the adipose tissue influenced the fat storage capacity of the adipocyte, whereas lipin levels in skeletal muscle acted as a determinant of whole-body energy expenditure and fat utilisation. Phan and Reue [4] concluded that variations in lipin levels alone are sufficient to induce extreme states of adiposity and may represent a mechanism by which adipose tissue and skeletal muscle modulate fat mass and energy balance.

Suviolahti et al. [5] analysed lipin mRNA levels in mouse and human adipose tissues and observed both a strong negative correlation between lipin mRNA levels and fasting glucose and insulin levels. They genotyped seven lipin-1 gene (*LPIN1*) single nucleotide polymorphisms (SNPs) in 1109 individuals from 92 dyslipidemic Finnish families and in a case-control sample of 477 obese and 821 lean individuals, and identified SNPs and allelic haplotypes associated with serum insulin levels and body mass index. They concluded that lipin plays a role in glucose homeostasis and suggested that variation in *LPIN1* has significance in human metabolic traits.

Of the three lipins, lipin-1 is normally expressed at high levels in adipose tissue and skeletal muscle, lipin-2 is mostly expressed in the liver and brain, whereas lipin-3 is expressed in the small intestine and liver [2,3]. In human mammary epithelial cells, expression of *LPIN1* and *LPIN2* increased (*p* < 0.01) 4- and 2-fold, respectively, by day 4 of lactation, suggesting they play a potential role in lactation too [6].

Lipin-1 may also act as a co-regulator of transcription factors [2]. It does not itself have a DNA-binding motif, so its involvement in transcriptional regulation is probably through its transcriptional activating domain (TAD), which is located between residues 217 and 399 at the N-terminal [7]. The TAD domain does not show high sequence homology to lipin-2 and lipin-3 and appears to be unique to lipin-1, and it only interacts with peroxisome proliferator-activated receptor gamma (PPARγ), but not peroxisome proliferator-activated receptor alpha (PPARα). This allows the transcriptional co-activation of adipogenesis to be separated from the co-activation of genes involved in fatty acid oxidation [7].

Most of the reported sequences for human *LPIN1* are derived from mRNA sequences and vary in length. The gene structure is not well described, albeit Cao and Hegele [8] suggest humans have 21 exons. Péterfy et al. [9] report that two major isoforms of lipin 1 exist; lipin-1α and lipin-1β, and that lipin-1α is an 890 amino acid protein and lipin-1β is 924 amino acids in length. Each exhibits a distinct expression pattern, subcellular localisation, and cellular function [9]. Lipin 1β differs from lipin 1α in containing an alternatively spliced exon of 99 bp that is located in intron 4 [9]. Over 35 lipin-1-deficiencies in humans have been reported [10,11,12,13] and over 20 *LPIN1* mutations have been described in several ethnic groups, but no clear genotype-phenotype relationship has been revealed [10,11,12,14]. In mice, a mutation p.Ser724Leu stops all phosphatidate phosphatase activity [15], and this along with the human evidence, suggests that small changes in *LPIN1* have substantive effects functionally.

The cattle *LPIN1* gene (ENSBTAG00000007689) is on chromosome 11 and has four transcripts (*LPIN1-201*–*LPIN1-204*) described in the Ensembl database, that would produce proteins that varied in size from 900 to 935 amino acids in length. These length differences appear to come about because of variation in splicing at the 5′-end of the gene, but with Ensembl describing 21 potential exons, with the ATG start site in exon 2. GenBank (https://www.ncbi.nlm.nih.gov/gene/537224; accessed on 2 November 2021) contains 13 bovine *LPIN1* sequences, but describes 28 exons and seven isoforms, with the variation once again being at the 5′-end of the gene.

In dairy cows, Bionaz et al. [16] reported that all three lipins were expressed in the mammary gland, and that the expression of lipin-3 dominated at the end of pregnancy, whereas the expression of lipin-1 and lipin-2 predominated during lactation with the largest increase in expression being observed for lipin-1. In lactating Chinese Holstein cows, Han et al. [17] found *LPIN1* to be highly expressed in the mammary gland and liver and reported that SNPs in *LPIN1* were associated with milk yield, fat yield, fat percentage, and protein yield. In Holstein cattle, Li et al. [18] investigated gene expression in the liver of Holstein cattle during their dry period, in early lactation and at the peak of lactation, and identified *LPIN1* as a ‘promising candidate’ affecting milk yield, milk protein and milk fat traits.

In yaks, variation in the 5′ non-coding region of *LPIN1* has been reported to associate with milk fat content and milk solids [19]. In Brown Swiss cows, Pegolo et al. [20] reported that SNPs in *LPIN1* affected milk fatty acid composition, but Nafikov et al. [21] did not find any association between *LPIN1* variation and milk fatty acid composition in Holstein cows. In Chinese Holstein cows, Han et al. [17] reported associations of variation in *LPIN1* with milk yield, milk fat percentage and milk protein percentage, but some of those associations were only detected for only the first or second lactations. Together, these findings suggest that *LPIN1* may have an effect on the regulation on milk traits, but more investigations are needed to confirm whether the effect is robust, or whether it varies between breeds and/or varies depending on how the cattle are farmed.

New Zealand (NZ) is a major dairy producer and global exporter, with cows predominantly farmed outdoors on pasture. Milk production is seasonal, with exports predominantly being dry powders, and cheeses and butter, but rarely whole milk. Accordingly increasing milk solid production is a priority for the industry. The Holstein-Friesian × Jersey (HF × J)-cross or Kiwicross^TM^ cow is now the most common dairy cow in NZ. It was created to enable a variety of benefits, including a reduction in cow frame size and weight that is advantageous for maintaining soil structure (reducing compaction) when being grazed on pasture, and having a lower feed maintenance cost (because of its reduced weight) than the larger HF cattle. The cross is also considered to have better udders, improved longevity, increased calving ease, increased milk fat production, and a gestation period that is typically shorter than HF-type cattle.

In the 2019–2020 season, 49.1% of NZ dairy cattle were HF × J-cross, with only 32.7% being HF and 8.4% being Jersey cows [22]. In this study, the effect of *LPIN1* variation on milk traits was investigated in HF × J-cross cows that were run in a wholly pasture-based outdoor dairy production system. Variation in *LPIN1* was analysed using PCR-single strand conformation polymorphism and DNA sequencing and three gene regions were chosen for analysis. These were a portion of the bovine *LPIN1* 5′ non-coding region that corresponds to a region which has been associated with milk traits in yaks [19], the *LPIN1β*-spliced exon that excision/inclusion may affect gene expression or protein function, and the sixth coding exon that encodes the TAD domain, which is involved in the transcriptional regulation of other genes. The overall purpose of the research was to ascertain whether variation in *LPIN1* genotype could be utilised to select and breed dairy cattle for improved milk solid production.

## 2. Materials and Methods

### 2.1. Cows Investigated and Sample Collection

The Lincoln University Animal Ethics Committee (AEC Number 521) approved this research under the provisions of the Animal Welfare Act 1999 (New Zealand Government).

A total of 454 Holstein-Friesian × Jersey (HF × J)-cross dairy cows with ages ranging from 3 to 10 were investigated. All the cows were grazed outdoors on pasture (a mixture of perennial ryegrass and white clover) and sourced from two different herds at the Lincoln University Dairy Farm, NZ. All the cows calved over the months August–September, and they were milked twice per day. Over the period of milk sample collection, they were grazed at between 4 and 4.3 cows per hectare.

Udder milk samples for milk trait analyses were collected once a month from September to February, and the daily milk yield in litres from each individual cow was recorded using Tru-test in-line milk meters (Tru-test Ltd., Auckland, New Zealand). The milk samples were analysed for fat percentage (%) and protein percentage (%) using Fourier-Transform Infra-Red Spectroscopy (MilkoScan FT 120, Foss, Hillerød, Denmark).

A blood sample from each of the cows was collected onto FTA^TM^ (Cytiva, Marlborough, MA, USA) cards and air dried. Genomic DNA for PCR amplification was purified from a 1.2 mm punch of the dried blood spot using a two-step washing procedure as described by Zhou et al. [23].

### 2.2. Variation Screening and Genotyping

Blood samples (*n* = 132) were randomly selected to screen for nucleotide sequence variation in three regions of bovine *LPIN1*: a portion of the 5′ non-coding region, a region containing the *LPIN1β*-spliced exon, and the sixth coding exon. Three pairs of PCR primers (Table 1) for these regions were designed based on the cattle genome assembly sequence (NC_037338.1), and the primers were synthesised by Integrated DNA Technologies (Coralville, IA, USA).

The PCR amplifications were performed as 15 µL reactions containing the purified genomic DNA on a 1.2 mm punch of the washed FTA^TM^ paper, 0.25 µM of each set of paired primers, 150 µM of each dNTP (Bioline, London, UK), 2.5 mM of Mg^2+^, 0.5 U of Taq DNA polymerase (Qiagen, Hilden, Germany), and 1× the reaction buffer supplied with the DNA polymerase enzyme.

The amplifications were undertaken using S1000 thermal cyclers (Bio-Red, Hercules, CA, USA) and the thermal profile included an initial denaturation for 2 min at 94 °C; followed by 35 cycles of 30 s at 94 °C, 30 s at 58 °C and 30 s at 72 °C; with a final extension for 5 min at 72 °C.

A 0.7 µL aliquot of the PCR products was mixed with 7 µL of loading dye (98% formamide, 10 mM EDTA, 0.025% bromophenol blue, 0.025% xylene-cyanol). After denaturation at 95 °C for 5 min and immediate cooling on wet ice, the samples were loaded on 16 × 18 cm acrylamide:bisacrylamide (37.5:1) (Bio-Rad) gels and electrophoresed in 0.5× TBE buffer for 19 h using Protean II xi cells (Bio-Rad), under the conditions described in Table 1. The gels were silver-stained using the method described by Byun et al. [24].

When the most variable region of *LPIN1* had been found, all 454 cows were then typed using the PCR-SSCP technique described above.

### 2.3. Sequencing of Variants and Sequence Analysis

Homozygous PCR amplicons identified using PCR-SSCP were sequenced at the Lincoln University DNA Sequencing Facility. If there were no homozygous samples that could be sequenced directly, the variants were sequenced using an approach described previously [25]. In this approach, single bands of interest from the PCR-SSCP gels for heterozygous cattle were recovered directly from the gels as a thin slice. This slice was macerated and the DNA was eluted into 50 µL TE buffer by incubating at 70 °C for 20 min. The original primers and 1 µL of the eluted solution (as a template) were used for a second round of PCR amplification to produce a simple SSCP gel pattern that could be directly compared to the pattern derived from the original heterogeneous amplicons. The second ‘homozygous’ PCR amplicons were then sequenced at the Lincoln University DNA Sequencing Facility.

Sequence alignment and comparisons were carried out using DNAMAN (version 5.2.10, Lynnon BioSoft, Quebec, QC, Canada).

### 2.4. Statistical Analyses

Statistical analyses were carried out using IBM SPSS version 22 (IBM, New York, NY, USA). Generalized linear mixed models (GLMMs) were used to test associations between the presence or absence of individual variants of *LPIN1* and variation in milk traits. Cow age in years, herd, and the number of days in milk (DIM) from the start of lactation were included in the models. The effect of cow sire could not be included in the GLMMs as some semen straws (sire genetics) used in NZ dairy cattle artificial insemination-based breeding approaches contained mixed-sire semen purchased from commercial semen producers. In these cases, individual sire identity is impossible to ascertain, but because the straws were mixed-semen straws and because different sires are used for different inseminations in different years, it is unlikely that sire in these cows was a strongly confounding effect. Cow age and herd might also be confounded with sire in these cows, but this cannot be confirmed.

## 3. Results

**Figure 1:**
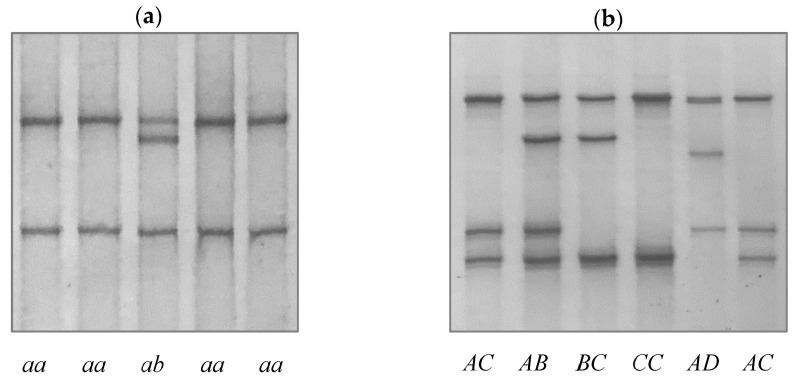
Sequence variants of bovine *LPIN1* identified by PCR-SSCP analysis. Two variants (*a* and *b*) in the 5′ non-coding region (**a**) and four variants (*A* to *D*) in the sixth coding exon (**b**) are shown in either a homozygous or heterozygous form, with the genotypes being labelled under the corresponding banding patterns.

Two PCR-SSCP banding patterns (*a* and *b*) were detected in the 5′ non-coding region of bovine *LPIN1* (Figure 1a). Sequencing of PCR amplicons for these two banding patterns revealed two sequence variants resulted from a SNP c.-30712C/T, with sequence variant *a* being identical to the cattle genome assembly sequence NC_037338.1.

The PCR-SSCP analysis of the *LPIN1β*-spliced exon region did not reveal any noticeable difference in banding pattern, and only one pattern was consistently obtained under various SSCP conditions including variations of polyacrylamide gel concentration, electrophoresis temperature and voltage. This suggests that sequence variation, if present in this region, could not be detected in these cows.

**Figure 2:**
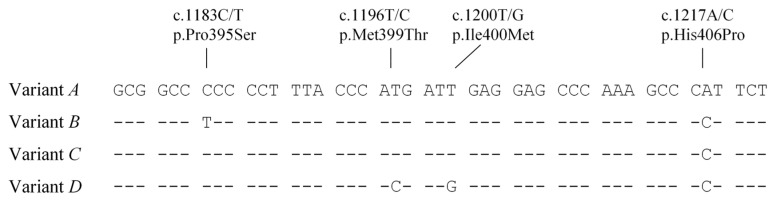
Sequence variation in the sixth coding exon of bovine *LPIN1*. Four variant sequences (*A* to *D*) containing four non-synonymous single nucleotide polymorphisms are identified and only the variable regions of the sequences are shown. Dashes represent nucleotide sequences identical to the top sequence.

For the sixth coding exon (noting that this may not be exon 6, as the cap site of the mRNA has not been described in detail, but the location of the ATG start site is known), four PCR-SSCP patterns representing four sequence variants (*A* to *D*) were detected (Figure 1b). There were four SNPs (c.1183C/T, c.1196T/C, c.1200T/G, and c.1217A/C) identified among these variants, which if transcribed and translated would result in the amino acid changes p.Pro395Ser, p.Met399Thr, p.Ile400Met, and p.His406Pro respectively. All of these SNPs were clustered close together (Figure 2) and have been reported in the Ensembl SNP database with reference numbers rs207681322, rs44114138, rs476118552, and rs137642654, respectively. Variant *A* had a sequence identical to the cattle genome assembly sequence NC_037338.1.

The effect of sequence variation in the sixth coding exon on milk traits was subsequently investigated in 454 NZ cows. A total of nine different genotypes were found: *AA* (*n* = 54), *AB* (*n* = 63), *AC* (*n* = 147), *AD* (*n* = 6), *BB* (*n* = 17), *BC* (*n* = 84), *BD* (*n* = 3), *CC* (*n* = 76), and *CD* (*n* = 4). This gave variant frequencies of 35.7%, 20.3%, 42.6%, and 1.4% for *A*, *B*, *C,* and *D*, respectively.

As variant *D* was present at a frequency of less than 5.0% in this study, cows carrying this variant were removed from the association study, as the small sample size might adversely bias the analyses. The presence of variant *C* was found to be associated with an increase in milk fat percentage (presence 4.96 ± 0.034% vs. absence 4.81 ± 0.050%; *p* = 0.006) and milk protein percentage (presence 4.09 ± 0.017% vs. absence 3.99 ± 0.025%; *p* = 0.001) (Table 2). No associations were detected for other variants.

## 4. Discussion

This study investigated genetic variation in three regions of bovine *LPIN1* and reported varying levels of variation in these regions. The absence of variation within the bovine *LPIN1β*-spliced exon region in this study is consistent with that reported by Nafikov et al. [21] and Han et al. [17]. Nafikov et al. [21] sequenced 12 unrelated Holstein cows from the Iowa State University herd and Han et al. [17] sequenced 1067 Chinese Holstein cows that belonged to 40 sire families from 22 dairy farms, and both of them did not find any variation in this region. However, variation in this region has been reported in sheep [26]. This suggests that the sequence of this region may be conserved in cattle and that species-based differences in the gene may occur.

In the sixth coding exon, four nucleotide sequence variants were analysed, and these contained four SNPs in the NZ Holstein-Friesian × Jersey (HF × J)-cross cattle analysed. However, only two [c.1183C/T (p.Pro395Ser) and c.1217A/C (p.His406Pro)] of these SNPs were reported in the Iowa State University Holstein cows with the amino acid changes being labelled as Pro396Ser and His407Pro by Nafikov et al. [21]. Why the numbers differ by one amino acid is unknown as the reading frame is correct for the known peptide sequence and ATG site. In the Chinese Holstein cows that Han et al. [17] studied, these were labelled as c.1521C>T (p.Pro398Ser) and c.1555A>C (p.His409Pro), and while the amino acid changes appear to be consistent with our finding, and those of Nafikov et al. [21] with respect to the amino acid changes and distance between the SNPs, the nucleotide positions are hugely disparate. It should also be noted that these two SNPs were linked and were only detected in variant *D*. This variant may be very rare in Holstein cattle, or it may be absent in Holstein cattle and have originated from the Friesian and/or Jersey breeds.

All four of the SNPs were non-synonymous, and three of the amino acid substitutions would result in changes in side-chain polarity. Given that this region encodes for the putative transcriptional activating domain, these amino acid substitutions may affect its interaction with PPARγ and consequently affect the transcriptional regulation of genes.

Of the three common sixth coding region variants (*A*, *B*, and *C*), associations with milk traits were only detected for variant *C*, but not variants *A* and *B*. Variant *C* only differed to *A* at SNP c.1183, and only differed to *B* at SNP c.1217. The associations detected in this study cannot be explained by either SNP in isolation alone, but instead must be a consequence of the linkage of these two SNPs (i.e., the SNP haplotype, or whole sequence of the variant). This is because an association analysis of the variation defined by the SNP c.1183, would be to compare cows carrying variant *B* to cows carrying variants *A* and *C*, and an association analysis of the variation defined by SNP c.1217 would be to compare cows carrying variant *A* to cows carrying variants *B* and *C*. This highlights the importance of SNP haplotypes, the whole variant sequence amplified, or even the regions outside of the amplified sequence, which are nevertheless in linkage with the SNPs, in the association analysis.

Direct determination of the SNP haplotypes or variant sequences revealed here would be difficult for direct DNA sequencing or with SNP chip detection approaches. In comparison, the PCR-SSCP approach is both simple and reliable. It is only limited by the length of the DNA fragments that can be easily analysed, with good resolution being typically observed for PCR-amplified fragments of under 600 bp in length [27,28,29,30].

The sequences of the three variants (*A*, *B*, and *C*) reported in this study would correspond to the haplotypes H1, H2, and H3 of the *LPIN1*-*2* block described in Han et al. [17]. They reported that the haplotypes of the *LPIN1-2* block were associated with milk yield and milk protein yield, but not associated with milk fat yield, fat percentage, and protein percentage in the first and second lactations. These associations appear to be different to those revealed here. What is more, in the study of Han et al. [17], cows with the H3 haplotype of *LPIN1-2* appeared to have a lower milk protein yield, whereas in our study, the corresponding variant (*C*) was found to be associated with increased synthesis of milk proteins. It is unknown whether these discrepancies reflect differences in cow age, feed, or breed, or whether the two extended haplotypes, while similar in the region of the SNPs, were nevertheless functionally dissimilar elsewhere.

The findings that *LPIN1* variation was associated with milk fat percentage and protein percentage, but not with milk yield, suggest that lipin-1 affects the synthesis of milk fat and milk proteins. Lipin-1 is a phosphatidate phosphatase enzyme required for lipid synthesis and also acts as a transcriptional coactivator of lipid metabolism genes. Its regulatory role in the lipid metabolism is well accepted [31]. The regulation of milk protein synthesis appears to be more conserved than the regulation of milk fat synthesis, as milk protein synthesis seems to be primarily regulated at the post-transcriptional level, rather than the transcriptional level, with the insulin-mammalian target of rapamycin (mTOR) pathway playing a central role [32]. In mice, the mTOR complex 1 has been shown to directly phosphorylate lipin-1 to regulate its localisation and nuclear eccentricity, and the effects of the mTOR complex 1 on the sterol regulatory element binding protein (SREBP) pathway and lipid homeostasis is mediated by lipin-1 [33]. It is accordingly very likely that lipin-1 is involved in the mTOR pathway and regulates protein synthesis.

## 5. Conclusions

Lipin-1 plays a regulatory role in tissues that function in lipid metabolism. In dairy cows, the lipin-1 gene is highly expressed in the mammary gland, but its function in milk production is less understood. In this study, we used PCR-single strand conformation polymorphism to reveal four sequence variants containing four non-synonymous SNPs in the sixth coding exon. Variant *C* was found to be associated with an increase in milk fat percentage and milk protein percentage, but no association was detected with milk yield. These results suggest that variation in *LPIN1* has potential as a breeding gene-marker to improve milk solid production traits.

## Figures and Tables

**Table 1:** PCR-SSCP conditions for the amplification and PCR-SSCP analysis of three regions of bovine *LPIN1.*

Region Amplified	PCR Primer Sequences (5′–3′)	Amplicon Size	SSCP Conditions
5′ non-coding	ACAAGGAGAGAACATGGGAG	416 bp	14% gel, 350 V, 16 °C
	CACACCTCAGCACTGGGTC		
*LPIN1β*-spliced exon	AGCAATTCACTATGGGCCTGC	468 bp	10–14% gel, 250–390 V, 4–25 °C
	CACATAAGTAATTTGGTTAATGG		
6th coding exon	GATCCAGTCCTCACCACAC	443 bp	10% gel, 390 V, 12 °C
	CAAGAGAGATGTCCTGTCTC		

**Table 2:** Association between the presence or absence of individual variants in the *LPIN1* sixth coding exon and milk traits ^1^.

Milk Trait	Variant	Absent	*n*	Present	*n*	*p*-Value
		Mean ± SE		Mean ± SE		
Milk yield (L/d)	*A*	21.81 ± 0.269	177	22.10 ± 0.232	264	0.384
	*B*	22.04 ± 0.222	277	21.88 ± 0.285	164	0.643
	*C*	22.28 ± 0.311	134	21.86 ± 0.211	307	0.228
Milk fat percentage (%)	*A*	4.93 ± 0.044	177	4.91 ± 0.038	264	0.666
	*B*	4.92 ± 0.036	277	4.92 ± 0.046	164	0.989
	*C*	**4.81 ± 0.050**	134	**4.96 ± 0.034**	307	**0.006**
Milk protein percentage (%)	*A*	4.08 ± 0.022	177	4.05 ± 0.019	264	0.169
	*B*	4.06 ± 0.018	277	4.07 ± 0.023	164	0.812
	*C*	**3.99 ± 0.025**	134	**4.09 ± 0.017**	307	**0.001**

^1^ Predicted means and standard error of those means were derived from the GLMMs. ‘Cow age’, ‘days in milk (DIM)’ and ‘herd’ were fitted as fixed effects, with *p* < 0.05 being shown in bold.

## Data Availability

The data presented in this study are available on request from the corresponding author.
